# Incisional Hernia Repair During Cesarean Section

**DOI:** 10.7759/cureus.24121

**Published:** 2022-04-13

**Authors:** Fatin A Aylia, Samir Khirie, David Steinberg

**Affiliations:** 1 Obstetrics and Gynecology, California Hospital Medical Center, Los Angeles, USA

**Keywords:** surgical treatments of hernias, hernia repair during c-section, maternal obesity, cesarean birth, abdomen ventral hernia

## Abstract

This case report follows the treatment of a 32-year-old Hispanic female who developed an incisional hernia after her first cesarean delivery. During her second cesarean section, the ventral hernia needed to be repaired due to the inability to approximate the fascial ring and close the abdominal wall. Hernias, in general, are uncommon during pregnancy and given that ventral hernias are virtually nonexistent in this patient population, we are left to deal with a host of different obstacles in their diagnosis and treatment.

## Introduction

Ventral hernias, often associated with incisional hernias, are defects such as a bulge or protrusion of abdominal contents through a weak point in the abdominal wall. Risk factors include any prior incision within the abdominal fascia or any pathology that causes increased intra-abdominal pressure such as chronic cough, lifting weights, constipation, and weight gain/obesity, as well as pregnancy [1]. The recurrence rate of ventral incisional hernias following repair can be as high as 15-40% [2]. This recurrence is dependent and influenced by obesity and lifestyle modifications of the patient. We present the outcome of a single case of ventral hernia repair during an elective C-section in a 32-year-old female.

## Case presentation

A 32-year-old G3P1 patient delivered her second child at a gestational age of 39 1/7 weeks via repeat low transverse cesarean section with vacuum assistance due to infant macrosomia. She had a ventral hernia that complicated her pregnancy course. The hernia was located in the left middle quadrant of the patient’s abdomen and measured approximately 12 cm. The associated risk factors for this patient include an elevated BMI of 52.33 kg/m^2^ and a previous surgical history of low transverse cesarean section during her first pregnancy.

After a Pfannenstiel skin incision was made, the superficial boundary of the hernia sac/peritoneum was evident just under the adipose layer. Following the delivery of the baby and closure of the uterus, there was a large hernia sac but the fascia was not clearly identified other than a deep, tight ring that was palpable at the base of the hernia sac. The general/trauma surgeon was called in to assist in closing the fascia. The primary closure of the fascia ring was done with interrupted sutures. After the fascia was reapproximated, the hernia sac was then trimmed and a Jackson-Pratt surgical drain was placed over the fascia to eliminate fluid collection in the location of the old hernia. After irrigation, the subcutaneous layer was then reapproximated with a two-layer closure.

The patient’s postoperative course was uncomplicated. There were no complications during her two-week and six-week follow-ups with the general surgeon and her obstetrician-gynecologist, respectively.

## Discussion

Currently, there is no consensus on the optimal timing or treatment technique for a gravida with a large or symptomatic hernia [3]. The lack of an agreement on the treatment of ventral hernias in pregnant women makes the decision to treat and the process challenging. Treatment significantly depends on two factors: symptoms and pregnancy status at diagnosis. If the patient presents with a symptomatic hernia, meaning that it is either incarcerated (hernia is irreducible) or strangulated (constriction/ischemia of bowel), emergency repair is mandatory. However, if there is an asymptomatic hernia in a pregnant patient, the repair is often delayed until completed childbearing in order to decrease recurrence in future pregnancies. However, if the patient is suffering from symptoms and unhappy with their quality of life due to her symptoms, a repair can be done during pregnancy but postponed until at least after the second trimester when organogenesis is completed [3-4].

There are four avenues for the surgical repair of a ventral hernia: repair can be done either laparoscopically or via open approach with mesh repair or primary tissue repair with sutures, the latter of which was performed in this case. By combining the hernia repair and cesarean delivery, the patient would avoid a second incision, a second hospital stay, and a second risk for infection [5]. Combining both procedures is a safe and effective approach. The likelihood that post-cesarean section hernias will become incarcerated and strangulated in the future is influenced and positively correlated with an individual’s body habitus. Our patient was obese with a BMI of 52.33 kg/m^2^, which may put the patient at risk for recurrence in the future. There was no indication or plan to repair the hernia prior to beginning the c-section; however, the difficulty of closing the fascia due to the hernia forced its repair and a general surgeon was called to assist in the repair of the hernia. It was imperative to close the fascia in order to allow for proper healing of the abdomen. The fascia provides structural support to the abdomen and, if it was not properly repaired, could lead to a seroma, hematoma, or wound dehiscence. After the procedure, the patient was counseled on lifestyle modifications in order to avoid the recurrence of a ventral hernia.

## Conclusions

Here, we present a case of an incisional hernia repair using a primary tissue repair technique immediately following cesarean delivery. The advantages of concomitant repair of the hernia at the time of delivery may include avoiding longer operating times, a secondary incision, and hospital stay, decreasing the risk of hospital-acquired infections, and decreasing recovery time overall. Combining both procedures is ideal in patients who have completed childbearing, as this would decrease the risk of recurrence and the development of other hernias. However, in our case, there was no option to delay the repair of the hernia due to the degree of difficulty noted in closing the abdominal fascia in a way that would allow for proper healing and reduce the chances of developing a new hernia or seroma. In addition, it is of note to mention that patients with similar risk factors would likely benefit from lifestyle changes, including healthier diets, nutrition, weight loss, and exercise regimens implemented to prevent initial and recurring instances of ventral hernias and associated complications.
